# COVID-19: Infection or Autoimmunity

**DOI:** 10.3389/fimmu.2020.02055

**Published:** 2020-09-11

**Authors:** Timothy Icenogle

**Affiliations:** Tim Icenogle MD, PLLC, Spokane, WA, United States

**Keywords:** COVID-19, autoimmunity, myocarditis, hemophagocytic lymphohistiocytosis, thymoglobulin, rituximab, immunosuppression

## Abstract

The clinical and laboratory features of COVID-19 are reviewed with attention to the immunologic manifestations of the disease. Recent COVID-19 publications describe a variety of clinical presentations including an asymptomatic state, pneumonia, a hemophagocytic lymphohistiocytosis like syndrome, Multisystem Inflammatory Syndrome in Children (MIS-C) but, also called Pediatric Inflammatory Multisystem Syndrome-Toxic Shock (PIMS-TS), Kawasaki Disease, and myocarditis. A common theme amongst multiple reports suggests an overexuberant autoimmune component of the disease but a common pathophysiology to explain the variations in clinical presentation has been elusive. Review of the basic science of other viral induced autoimmune disorders may give clues as to why immunosuppressive and immunomodulating regimens now appear to have some efficacy in COVID-19. Review of the immunopathology also reveals other therapies that have yet to be explored. There is potential use of T cell depleting therapies and possibly anti-CD20 therapy for COVID-19 and clinical research using these medications is warranted.

## Introduction

The SARS-CoV-2 virus, as of July 21, 2020, has infected over 14 million people worldwide and resulted in over 600,000 deaths (1). It has spread to all continents except Antarctica and continues to spread. The current United States mortality rate is 3.7% (1).

The clinical features are quite variable and a common pathophysiology of this disease to explain the variation in the clinical features has yet to be described. The virus can directly invade cells of the upper and lower respiratory tree via its receptor binding domain by attaching to the ACE2 receptor on those cells. This is facilitated by TMPRSS2, to initiate the viral infection (2). Fortunately, most cases, 81%, are mild and self-resolve (3). In one series approximately 14% were classified as severe (dyspnea, respiratory frequency >30/min, blood arterial oxygenation <93%, partial pressure of arterial oxygen to fraction of inspired oxygen ratio <300, and/or infiltrates >50% within 24–48 h), and 5% are critical (i.e., respiratory failure, septic shock, and/or multiple organ dysfunction or failure) (3). The most common presenting symptoms to the hospital were fever, shortness of breath, expectoration, fatigue, dry cough and myalgia (4), symptoms suggestive of viral pneumonia (5). Of the patients with severe disease, they were noted to be older, male, and had other comorbidities (3, 4). In the more severely ill patients there was elevation of inflammatory cytokines suggestive of immune dysregulation (2, 4, 5) and possible autoimmunity. A review of other viral induced autoimmune disorders with particular attention to viral induced secondary hemophagocytic lymphohistiocytosis (sHLH), and the related syndromes of Macrophage Activation Syndrome (MAS), Multisystem Inflammatory Syndrome in Children (MIS-C) which is also called Pediatric Multisystem Inflammatory Syndrome -Toxic Shock (PIMS-TS), Kawasaki Disease, and myocarditis will hopefully lead to greater understanding. Type IV and Type II hypersensitivity autoimmune responses secondary to COVID-19 are compared with the Type IV and Type II responses seen in other viral induced autoimmune diseases. Studies in the immunology literature point to a different pathophysiology than that accepted by some clinicians but explain the partial efficacy of the immunosuppressive regimens used to date. The potential use of other therapies based on immunology and clinical research may prove to be more efficacious in COVID-19.

## Infection or Autoimmunity

The overexuberant immune response seen in COVID-19 raises the question as to the pathophysiology of the disease: Is the lethality related to an infection with the SARS-CoV-2 virus or to an uncontrolled autoimmune response induced by the virus, or perhaps, both? Viruses can induce Type II and Type IV hypersensitivity reactions in addition to a viral cytopathic effect.

Type II hypersensitivity occurs when autoantibodies secondary to the viral infection cause tissue damage. Type IV Hypersensitivity reactions occur when T cells primed to fight the viral infection induce inflammation or directly kill target cells of the host. These reactions can occur even if the virus has little or no cytopathic effect.

## Clinical Features and Pathophysiology of COVID-19

The clinical features of the disease and pathophysiology are areas of ongoing study. Pathologic analyses of patients with severe COVID-19 disease and SARS reveal a complex overexuberant inflammatory response (4–7). ICU patients with COVID- 19 had higher plasma levels of IL2, IL7, IL10, GCSF, IP10, MCP1, MIP1A, and TNFα compared to non-ICU patients with COVID-19 (5). COVID-19 patients were noted to have high amounts of IL1B, IFNγ, IP10 and MCP1 which were leading to activation of T-helper-1 (Th1) responses and the resulting cytokine storm was associated with disease severity (5). Sixty- three percent of hospitalized patients had lymphocytopenia but another study that focused only on ICU patients documented an 85% incidence of lymphocytopenia (8). Those authors suggested that severity of the lymphocytopenia reflects the severity of the disease.

In a report of one patient who died, post-mortem biopsies noted that the pulmonary tissue resembled that seen in severe acute respiratory syndrome (SARS) and Middle Eastern respiratory syndrome (MERS) (9). Pulmonary findings included ARDS, edema, hyaline membranes, interstitial mononuclear infiltrates, multinucleated syncytial cells with atypical enlarged pneumocytes but no intranuclear or intracytoplasmic viral inclusions were seen. Flow cytometry performed on peripheral blood revealed that counts of CD4^+^ and CD8^+^ T cells were substantially reduced, but those present were highly activated as evidenced by high proportions of HLA-DR (CD3), and CD 28 double fractions present. There were also increased concentrations of CCR6 + and Th17 in the CD4^+^ T cells. CD8^+^ T cells had high concentrations of cytotoxic granules in which 31.6% of cells were perforin positive, 64.2% were granulysin positive and 30.5% were granulysin and perforin double positive. These findings implied an overactivation of T cells, manifested by the increase in Th17 and the high cytotoxicity of the CD8^+^ T cells (9).

A Chinese study of 49 patients admitted to a single institution sought to determine which factors were associated with the progression to severe disease (10). Univariate analysis revealed that comorbidity, age >50, lymphocyte counts <1,500/μL and serum ferritin >400 ng/ml were predictive of progression to severe disease. The findings of lymphopenia and hyperferritinemia are suggestive of secondary hemophagocytic lymphohistiocytosis (HLH) which was seen in both SARS and MERS patients (11–15). Severely ill COVID-19 patients frequently display signs of a cytokine storm, elevated ferritin (4, 16, 17), and lymphopenia and should be evaluated for secondary hemophagocytic lymphohistiocytosis (sHLH) (18).

### Secondary Hemophagocytic Lymphohistiocytosis

Secondary HLH is a syndrome characterized by excessive cytokine production, subsequent immune dysregulation, and tissue damage and is frequently associated with viral infections (19). It is differentiated from primary HLH which has a similar clinical presentation but is caused by genetic defects in natural killer cells (NK), and T cells and usually appears in children. The difference between the two can be confusing because the phenotypic presentation is the same, but the inciting pathophysiology is vastly different. Secondary HLH associated with viral syndromes was first reported by Risdall et al. (20). The dysregulated inflammatory response causes fever, hepatomegaly, splenomegaly, cytopenias (affecting one or more of three lineages in the peripheral blood), neutropenia, hypertriglyceridemia, hypofibrinogenemia, elevated ferritin, hemophagocytosis in bone marrow, spleen or lymph nodes, low or absent natural killer (NK)-cell activity, and elevated soluble CD 25 (interleukin [IL]-2 receptor) (21). Excessive cytokine production (cytokine storm), by macrophages, NK cells, and cytotoxic T lymphocytes (CTLs) is thought to be the primary mediator of tissue damage (21, 22). A number of viruses have been linked to secondary HLH including avian influenza A subtype H1N1, SARS-CoV, Ebstein Barr, and rotavirus (23).

The pathologic manifestation of secondary HLH is the result of the excessive and persistent activation of macrophages and T cells (22) but there is also impaired cytotoxic function by NK cells and CD8^+^ T cells (24). Secondary HLH tends to have less severe clinical manifestations than primary HLH, but mortality is still considerable. A murine model of HLH has clarified the pathophysiological steps that result in the persistent activation (24). In a normal immune system, antigen presenting cells process the infecting virus and present viral peptides to CD4^+^ and CD8^+^ T cells. The T cells proliferate and differentiate and produce cytokines that activate APCs, including macrophages, to further augment antigen presentation in a positive feed forward system. CD8^+^ T cells produce INFγ, which is a potent stimulator of macrophages, and CD8^+^ T cells can also lyse viral infected APCs via a perforin dependent mechanism. Perforin dependent mechanisms are also a negative regulator in this positive feed forward activating system. The murine model was deficient in perforin (pfp^–/–^), and was infected with lymphocytic choriomeningitic virus (LCMV), a non-cytopathic virus that is common in wild mice. In the wild type mice, the virus causes a transient fever, and all survive. After infection, the pfp^–/–^ mice developed prolonged fever, splenomegaly, pancytopenia, hypofibrinogenemia, hypertriglyceridemia, and all mice die within 2 weeks. Histologic features included periportal infiltrates in the liver, disorganized infiltrates of macrophages and activated lymphocytes in the spleen and lymph nodes. In the bone marrow, the CD68^+^ macrophages display MHC class II molecules, a sign of activation, and this correlated with a significant increase in IFNγ secreting CD8^+^ T cells compared to wild type mice. The pfp^–/–^ mice produce significantly elevated levels of TNF-α, IFNγ, IL-6, IL-10, IL-18, and M-CSF and INFα, like humans with sHLH. To determine which cell lines were responsible for the cytokine secretion, 6 days after infection, the pfp^–/–^ mice were injected with monoclonal antibodies to deplete CD4^+^, CD8^+,^ or NK 1.1 cells. Most of the mice injected with the anti-CD8^+^ antibody survived while those injected with the anti-CD4^+^ or anti-NK1.1 antibodies did not. Additionally, when double knockout mice deficient for T and B cells and pfp^–/–^ were inoculated with LCMV, all survived. These experiments indicated that CD8^+^ T cells were essential to develop sHLH. A series of neutralizing experiments were done to determine which cytokines were key in the pathophysiology. When INFγ was neutralized, most of the mice survived, while neutralization of the other cytokines mentioned above did not provide a survival benefit. IFNγ secretion was found to be more prolonged and elevated in the pfp^–/–^ mice than the wild type mice. When immune cells were studied *ex vivo* almost all the IFNγ^+^ cells were CD8^+^ T cells. When the pfp^–/–^ mice were depleted of CD8^+^ T cells, they had significantly lower levels of IFNγ. If INFγ was blocked in pfp^–/–^ mice 6 days after infection, the histiocytic infiltrates and the cytopenias did not develop. To study what was causing the excessive production of IFNγ, CD8^+^ T cells were assayed in the lymph nodes, spleen, liver, and bone marrow of the pfp^–/–^ and wild type mice after infection. The number of CD8^+^ T cells were similar in both groups but a LCMV antigen specific assay revealed that the number of antigen specific CD8^+^ T cells were elevated by two to fivefold in the pfp^–/–^ mice over the wild type mice. Antigen specific staining also revealed that the CD4^+^ T cells had similar elevations. While the population of antigen specific CD4^+^ and CD8^+^ T cells were elevated two to fivefold, the elevation of INFγ was elevated between 10 and 1,000-fold compared to the wild type mice. It was determined that the number of CD8^+^ T cells spontaneously producing IFNγ were elevated without stimulation. Further studies indicated that after 6 days from infection both the wild type and the pfp^–/–^ mice had significant levels of virus in their spleens, but the pfp^–/–^ mice had 10-fold higher levels of infectious virus. These data indicate that persistent viral presentation led to elevated INFγ production. Failure to clear the virus secondary to impaired cytotoxic function led to the overproduction of INFγ by CD8^+^ T cells. The study reveals that sHLH is a T cell driven process wherein failure to clear the virus leads to prolonged and excessive T cell stimulation which in turn drives other immunological processes. The study revealed two possible therapies for sHLH: CD8^+^ T cell depletion and blocking IFNγ function.

A study of 39 COVID-19 patients with pneumonia revealed that CD4^+^ and CD8^+^ T cells were low in the peripheral blood but have an increased capability to produce IL-17 *in vitro* compared to controls (7). IL-17 strengthens the immune response and activates neutrophils. Studies in a primate model show that during inflammatory states, the IL-17 producing CD8^+^ cells may be fourfold higher in the lung and the CD8^+^ cells in the lung can produce more IL-17 than the cells in the blood (25). Other cytokines were also increased in this population of COVID-19 patients. IFNγ, a Th1 molecule, was fourfold higher in COVID-19 patients as compared to controls despite the increased presence of markers of exhaustion and senescence and a skewing of cells toward TH17 phenotype. The study concluded that blockade the IL-17 pathway maybe efficacious in COVID-19.

A study of 33 patients using flow cytometry confirms the tendency for increasing lymphopenia in sicker COVID-19 patients who required hospital or ICU care (26). Immune cells and cytokines in peripheral blood were evaluated to determine factors related to the pathophysiology. CD4^+^ and CD8^+^ T cells are markedly reduced but there is no difference in the numbers of B cells, NK cells, or leukocytes between patients and controls. CD4^+^ T cells have indicators of activation and a subset of CD4^+^ and CD8^+^ T cells have indicators of exhaustion. Intracellular cytokine staining was performed for INFγ, TNFα, GM-CSF, and IL-6 and a high expression of GM-CSF^+^ and IL-6^+^ expressions were found in the CD4^+^ T cells. Pathogenic Th1 CD4^+^ T cells co-expressing IFNγ and GM-CSF were found only in the ICU patients indicating that these cells play an important role in the hyperinflammatory response of COVID-19. CD8^+^ T cells from ICU patients also showed a higher expression of GM-CSF compared to non-ICU or control patients. GM-CSF stimulates monocytes and the percentage of CD14^+^ and CD16^+^ monocytes was much higher in the severely ill patients in the ICU. These monocytes also can secrete GM-CSF and IL-6 to further enhance the inflammatory storm. The excessively activated immune response initiated by Th1 T cells and enhanced by CD 14^+^ and CD16^+^ monocytes may cause the pulmonary pathology of COVID-19. The authors suggest that IL-6 blockade may be helpful in COVID-19.

These studies all point to a prolonged and excessive activation of T cells that then leads to the T cell mediated damage in the sHLH presentation in COVID-19. While this is evidence that the pathophysiology of sHLH meets the definition of a Type IV hypersensitivity reaction, the etiology of that prolonged and excessive stimulation is an active area of study.

When viruses invade the host APCs and start to replicate, this initiates a cascade on antiviral defenses. The viral replication products, called viral pathogen associated molecular patterns (PAMPS), are recognized by host cell pattern recognition receptors (PRR), that activate transcription factors i.e., interferon regulator factors (IRFs), and nuclear factor 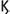
B (NF-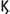
B). NF
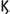
B leads to the production of pro-inflammatory cytokines while IRFs lead to the production of Type I and Type III interferons that are capable of inhibiting viral replication. The Type I interferons (IFN-α, IFN-β, IFN-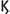, and IFN-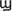), bind to the type I interferon receptor (IFNAR) at the plasma membrane in an autocrine and paracrine manner (27–29). A robust antiviral defense program initiates and hundreds of interferon-stimulated genes (ISGs), are produced which have the capability to interfere with every step of viral replication. Type III INFs (INF-λ), bind to the IFNLR receptor that is preferentially expressed on epithelial and some myeloid cells. Viruses have unfortunately developed mechanisms to avoid detection and to suppress the functions of IFNs and ISGs. Transcriptome profiling of certain cell types reveals that SARS-CoV-2 infection elicits exceptionally low INF-I and IFN-III and reduced ISG while inducing pro-inflammatory chemokine and cytokine genes (30). The SARS-CoV-2 virus has a non-structural protein (NSP16), that is over 92% identical to a protein in SARS-CoV-1 and it is able to hide the SARS-CoV viruses from the double stranded RNA pattern recognition receptors of the host cell. This protein system and others of the SARS-CoV-2 virus antagonize the production of the interferons that would otherwise inhibit viral replication. Studies have also shown that monocytes from older humans have defective IFN-I and IFN-III production while maintaining intact production of inflammatory cytokines (27). These factors may partially explain the poorer prognosis in older patients. The potential therapeutic uses of IFNs are discussed later but these studies infer that ineffective clearance of the virus results in prolonged replication and subsequent overstimulation of T cells.

The diagnosis of primary and secondary HLH is made based on both clinical and laboratory findings, none of which by themselves are diagnostic. Five of the following eight findings are diagnostic of HLH as outlined in the HLH-2004 guidelines (31, 32):

1. Fever ≥38.5°C.
2. Splenomegaly.
3. Peripheral Blood cytopenia, with at least two of the following: hemoglobin <9 g/dl; platelets <100,000/μL; absolute neutrophil count <1,000/μL.
4. Hypertriglyceridemia (fasting triglycerides >265 mg/dL).
5. Hemophagocytosis in bone marrow, spleen, lymph node or liver.
6. Low or absent NK cell activity.
7. Ferritin >500 ng/mL.
8. Elevated soluble CD 25 [soluble IL-2 receptor alpha (sIL-2R)] two standard deviations above age-adjusted lab specific normal.

The finding of hemophagocytosis is not necessary to make the diagnosis of HLH. Other findings include a histological picture of chronic persistent hepatitis, decreased fibrinogen, coagulopathies, and elevated levels of cytokines (IFN-γ, IL-10, and IL-6) (19).

It is sometimes difficult to diagnose HLH so a diagnostic score was developed called the HScore (32). The HScore can be used to estimate an individual’s risk of having hemophagocytic syndrome. The scoring system is available online at http://saintantoine.aphp.fr/score/ or in MedCalc^®^. These diagnostic tools are most often applied to pediatric patients with primary HLH but can be applied to patients with secondary HLH.

### Treatment of HLH and Application to COVID-19

Treatment for HLH is often directed to the pediatric patients with primary HLH. These patients have a genetic defect that results in HLH and the syndrome recurs unless the patient receives a stem cell transplant. The goal of therapy is to suppress life-threatening immune cells until the patient can receive the transplant (33). The standard therapy (HLH-94), consists of dexamethasone and etoposide and intrathecal methotrexate, and is intended as a bridge to transplant and produces a survival of 55% (34). A newer protocol for primary HLH (HLH-2004), added cyclosporin, a calcineurin inhibitor to the induction phase and hydrocortisone to the intrathecal methotrexate. The results of this trial are still pending (33). Steroids are known to induce apoptosis of T cells and suppress the production of cytokines. Steroids were initially thought to be contraindicated in COVID-19, but later reports revealed that dexamethasone reduced deaths by one-third in patients receiving mechanical ventilation (35). Etoposide is an anti-neoplastic agent that inhibits the cell cycle in the S phase or early G2 phase and is also a topoisomerase inhibitor.

Rabbit antithymocyte globulin (RATG), a T cell depletion therapy, was reported in a single center study of primary HLH with complete response in 73%, partial response in 24 and no response in one patient. The overall survival rate after stem cell transplant was 55%, comparable to HLH-94 (36). There are case reports of other therapies for refractory HLH which have included alemtuzumab, an anti-CD52 monoclonal antibody, anakinra, an IL-1 blocker, IVIG, and most recently, emapalumab, a INFγ inhibitor (33). Secondary HLH, despite the phenotypic similarities, is not the same disease as primary HLH and should not have the inevitable recurrence rate. There are no randomized trials in the literature for therapy of sHLH.

The HLH-94 protocol was used to treat secondary HLH in 126 patients without malignancy and 24 of these had an infection other than Ebstein-Barr virus (EBV) (37). Patients with an infection other than EBV had a 5 year survival rate of 78.7% demonstrating dramatically improved response over patients with primary HLH. There is an anecdotal report of a critically ill 31-year old man with secondary HLH due to influenza A/H1N1 who improved with etoposide and steroids in addition to antiviral therapy (38). This study emphasized that patients with viral induced secondary HLH should be considered for therapy only after they have met the diagnostic guidelines in HLH-2004 and that therapy was part of the HLH-94 protocol. The unique approach of these two papers to treat a critically ill patients with ongoing infections with powerful cytotoxic regimens aimed at modulating the immune system cannot be understated. The infectious agents initiated a severe autoimmune attack that was successfully treated with immunotherapy directed at a broad range of immunologically active cells. Treating an infection with powerful immunosuppression is a paradox with medical tradition and understanding. The role of antiviral agents in these scenarios is not yet proven, but it seems prudent to consider their use when using powerful immune suppression.

### Secondary Hemophagocytic Lymphohistiocytosis-Like Syndromes

Macrophage activation syndrome, MAS, is a type of secondary HLH that occurs in the presence of an autoimmune disease, frequently in systemic juvenile idiopathic arthritis (sJIA), and the death rate is as high as 20–30% (39, 40). Unlike viral induced sHLH, there is an underlying autoimmune disease already present that predisposes the patient to the syndrome. Many rheumatologists feel that MAS should be classified as sHLH as the clinical presentation and pathophysiology appears to be similar if not identical. Soluble IL-2 receptor alpha chain (sCD25), and sCD163 may be elevated and a defect in lymphocyte cytolytic activity is proposed as the major pathophysiology (41). The first line therapy for sJIA is NSAIDs but many patients require therapy with biologic agents such as anakinra, canakinumab or tocilizumab or disease modifying anti-rheumatic drugs (DMARDs). Patients with sJIA who develop MAS are frequently on immunosuppression at the time that they develop MAS, sometimes with the very agents that are being studied as a therapy for COVID-19, i.e., anakinra, canakinumab and tocilizumab. These biologic agents do not prevent MAS in sJIA patients, but the incidence of MAS does not seem to be increased in the sJIA patients who are already on them. MAS is a life-threatening condition with a significant mortality rate and the first line therapy is steroids followed by steroids plus cyclosporine. A report of two critically ill patients of MAS, unresponsive to steroid and cyclosporine, were treated with RATG with prompt improvement (42). T cell depletion therapy might be considered for other sHLH patients unresponsive to conventional treatment.

An outbreak in London of a Kawasaki-like disease in children was reported in early May of 2020 (43) and second report soon followed from northern Italy (44). The London report detailed an “unprecedented cluster” of eight children, six of whom were of Afro-Caribbean descent, who presented with hyperinflammatory shock with features of atypical Kawasaki disease. All initially tested negative for SARS-CoV-2 on bronchoalveolar lavage or nasopharyngeal aspirates. A common finding was bright coronary arteries on echocardiogram and elevation of CRP, procalcitonin, ferritin, triglycerides, and D-dimers. One child developed refractory shock, required extracorporeal mechanical oxygenation (ECMO), and then died from a large cerebrovascular infarct. The clinical picture suggested a hyperinflammatory syndrome with multiorgan involvement similar to Kawasaki disease shock syndrome (KDSS). Before the report could be published, 20 more patients with similar symptoms presented to the hospital. The northern Italy report was from Bergamo province at the epicenter of the COVID-19 epidemic (44). There was a 30-fold increase in the incidence of Kawasaki Disease (KD), per the American Heart Association 2017 criteria, compared to the previous 5 years. The AHA criteria include fever, conjunctivitis, changes in lips or oral cavity, laterocervical lymphadenopathy, polymorphic rash, erythema of the palms and soles, and induration of the hands or feet. Additional criteria included the elevation of erythrocyte sedimentation rate, CRP or both in the presence of anemia, thrombocytosis after 7 days of fever, hypoalbuminemia, hypertransaminasemia, leukocytosis, sterile pyuria or an echocardiogram showing coronary aneurysms. KD is usually a self-limiting, acute vasculitis of medium caliber vessels which nearly always affects children. In the acute phase, children may present with the hemodynamic instability of KDSS. Some patients may also have MAS. In the Italian experience, 50% of the patients had MAS with elevated ferritin, platelets *leq*181 × 10^9^/L, AST > 48 IU/L, Triglycerides *geq*156 mg/dl, and fibrinogen ≤360 mg/dl. Fifty percent of patients had KDSS, 60% had abnormal echocardiograms, 55% had elevated troponin and all patients had elevated proBNP. The average age on onset was 3.5 years. All patients were treated with IVIG, but 70% of patients had a Kobayashi score (45) of 5 or more and so were treated with steroids in addition to IVIG. All patients recovered but the clinical and laboratory findings were different from previous patients with KD seen at that center in the previous 5 years. The COVID-19 associated patients were older, had respiratory and gastrointestinal involvement, cardiovascular involvement, and meningeal signs. Laboratory signs in the COVID-19 patients showed leukopenia, marked lymphopenia, increased ferritin, and markers of myocarditis, similar to the adult patients with COVID-19. The patients were noted to have a more severe disease course with resistance to IVIG, biochemical evidence of MAS, and clinical signs of KDSS. Other reports soon followed which added increasing knowledge to the syndrome (46–52). The surge in cases seemed to follow 2–3 weeks after the peak in COVID-19 infection in the local area. Only about 25% required supplemental oxygen in contrast to the experience in adults. Most patients were treated with IVIG and if inflammatory markers persisted, steroids were added. Some patients were given anakinra (anti-IL-1), or tocilizumab (anti-IL-6). Patients with cardiogenic shock and/or myocardial compromise are at higher risk of death although the mortality rate seems quite low compared to the experience in adults. The disease has been labeled, “Pediatric Multisystem Inflammation Syndrome temporally associated with SARS-CoV-2 (PIMS-TS) in the United Kingdom and “Multisystem Inflammatory Syndrome in Children (MIS-C) in the United States. MIS-C has unique features of both a Type IV hypersensitivity reaction and a Type II hypersensitivity reaction. The sHLH and MAS signs and symptoms speak of a dysregulated T cell-initiated pathology consistent with Type IV hypersensitivity. Kawasaki disease has oligoclonal IgA plasma cells within the arteries of affected children (53, 54) consistent with a Type II hypersensitivity reaction. In comparison to adults, the Type IV hypersensitivity reactions i.e., sHLH and MAS, of MIS-C appear to be less severe, but the Type II hypersensitivity reaction of COVID-19 associated KD appears to be more severe than classical KD. Fortunately, the therapy for MIS-C, based on the therapy for KD, produces excellent results except for those with severe KDSS and myocarditis. In this subset of MIS-C patients perhaps a more aggressive therapy would be appropriate.

### Myocarditis

Evidence of myocarditis in COVID patients is evident from multiple studies (5, 8, 55–60). Cardiac injury as demonstrated by troponin I levels above the 99th percentile upper reference limit or new abnormalities on echocardiography or electrocardiography was seen in 12% of severely ill patients demonstrating virus related injury to other organ systems than the lungs (4). In a study of 416 hospitalized patients with COVID-19, 82 (19.7%) had evidence of myocardial injury evidenced by elevation of high-sensitivity troponin I (TnI) levels (57). A similar retrospective study of 187 hospitalized patients with COVID-19 disease revealed that 52 (27.8%) had myocardial injury by elevated levels of troponin T (TnT) (58). The patients with cardiac injury had higher rates of mortality and evidenced severe systemic inflammation with increased leukocyte counts, increased levels of C-reactive protein and procalcitonin. Review of the pathophysiology of viral induced myocarditis may provide clues to new therapies for COVID-19.

Woodruff first established that T cells have a critical role in the pathogenesis of myocarditis in 1974 using a mouse model (61). Coxsackie B_3_ virus (CBV3), which can cause myocarditis in humans, was injected into two mouse strains, CD-1 mice and Balb/c mice. In the CD-1 mice, the infection causes a systemic non-lethal reaction and viral replication is suppressed by day eight. Histologically, patchy infiltrates appear in the heart with macrophages and mononuclear cells on day 6, which resolves and fibroses by day 12. In the CD-1 mouse, pre-treatment with rabbit anti-thymocyte serum greatly suppressed the inflammation and tissue injury after CVB3 infection. In the Balb/c mouse, the infection is invariably lethal but viral replication stops by day 8. Histologically, cardiac myofiber necrosis and infiltration of mononuclear cells are prominent by day 6 and worsens over the next 2 days and the animal eventually dies. Infected Balb/C mice deprived of T cells by lethal irradiation and thymectomy had a decrease in inflammation, necrosis, and mortality. Viral growth curves in both strains were not different than normal immunologically intact animals, this led the investigators to conclude that the virus initiates the immune response and, despite high titers of virus in the heart, the T cells were the important mediators in the severity of inflammation and tissue injury.

Other investigators expanded on these observations. Huber showed that cytotoxic T cells from CVB3 infected mice would lyse virus infected myocardial cells *in vitro* independent of viral cytopathology (62). The Huber lab at the University of Vermont later showed that cytotoxic T lymphocytes derived from CVB3 infected mice were cytotoxic to uninfected Balb/c myocyte monolayers grown in culture. When T cells induce cardiac injury of uninfected cells, myocarditis becomes an autoimmune disease (63). Additionally, when control DBA/2 and Balb/c mice were infected with CVB3, they developed myocarditis, but if anti-thymocyte serum was administered immediately prior to and after inoculation, then myocarditis was prevented (64). From these studies and others, T cell depletion therapies seemed likely to have a reasonable probability for successful treatment of viral induced myocarditis. These papers firmly established viral induced autoimmune myocarditis as a Type IV hypersensitivity disease.

### T Cell Depleting Therapies in Myocarditis

Gilbert et al. reported the first use of a T cell depleting therapy for myocarditis in 1988 using OKT3. Muromonab-CD-3 (Orthoclone OKT3, Centocor Ortho Biotech Products, LP Raritan, NJ, United States) (65). This is a T cell depleting agent that is now discontinued in the United States, and was reported in over ten studies to have efficacy in human myocarditis (66). A viral prodrome was present in 68.8% of the patients in these studies. Full or partial recovery was seen in 82.5% of the patients. It should be noted that other immunosuppressives like corticosteroids, cyclophosphamide, cyclosporine, azathioprine and IVIG were also used and the dose of OKT3 was also variable. OKT3 was approved for transplantation but was associated with a cytokine release syndrome shortly after being administered. It was also associated with late occurring lymphomas so its use in transplantation gradually waned.

Icenogle et al. reported the first use with rabbit thymocyte globulin (RATG), a T cell depleting medication, in six patients with fulminant viral myocarditis and hemodynamic instability in 2004 (67). They were treated with either locally manufactured or with the commercial preparation, Thymoglobulin (Anti-Thymocyte Globulin [rabbit] intravenous administration Genzyme, Cambridge, MA, United States), once it became available. All patients received one gram of methylprednisolone at the time of RATG administration. All patients had pre and post treatment heart biopsies which showed resolution of the myocarditis, and five of the six survived, all without heart transplantation. Five years after the initial report there was a report with five cases of fulminant myocarditis treated with a similar polyclonal anti-T cell medication, Atgam [Lymphocyte Immune Globulin, Anti-Thymocyte Globulin (Equine) Sterile Solution. Pfizer, New York, NY, United States] that revealed similar results with the patients making rapid recovery (68).

### Pharmacology of RATG

Thymoglobulin (RATG) is produced by inoculating pathogen-free New Zealand rabbits with fresh human thymocytes. The thymocytes are derived from thymus tissue removed during pediatric cardiac surgery. The thymus lies anterior to the heart and blocks the surgeon’s view of the heart and so a portion is removed to allow visualization. After inoculation, the rabbits make a polyclonal antibody response to the human thymocytes and this is then purified and pasteurized.

There are a variety of cells in the human thymus, so Thymoglobulin has antibodies to numerous immunologically active cells, immune response antigens, adhesion and cell trafficking molecules, and molecules involved in heterogenous pathways (69).

RATG has anti-T-cell properties causing complement mediated T-cell death in peripheral blood and apoptosis in the spleen and lymph nodes (69). RATG also has antibodies to CD20, CD27, CD28 CD38, HLA Class I and Class II, and a host of adhesion molecules, i.e., CD11a/CD18 (LFA-1), CD49/CD29 (VLA-4), CD50 (ICAM-3), CD54 (ICAM-1), CD58 (LFA-3), CD102 (ICAM-2), CD195 (CCR5), and has demonstrated anti-B cell properties *in vitro* and *in vivo* (69–71).

The cytokine storm that accompanies some patients with COVID-19 and HLH changes the endothelium of blood vessels from an anti-adhesive to a pro-adhesive status. Selectins and integrins on the surfaces of lymphocytes and the endothelium are critical to these events (69). The antibodies in Thymoglobulin attach to these adhesion molecules and the antigen-antibody complex is then internalized by the cell. The internalization of the antigen-antibody complex, called modulation, is a major function of Thymoglobulin and the related pathway is then inhibited (down-modulation). The down modulation effect of Thymoglobulin has been demonstrated in the experimental animal in an ischemia-reperfusion experiment (72).

### Myocarditis and Immunosuppression

The papers using T cell depleting therapies focus on patients presenting with fulminant myocarditis and the use of these therapies is rarely mentioned in clinical reviews of myocarditis except in the treatment of giant cell myocarditis (73–76). No T cell depleting therapies have ever been part of a randomized prospective trial in myocarditis. While most reviews consider, “immunosuppression,” and mention the prospective randomized trials of steroids, cyclosporine, azathioprine, and IVIG, none of medications have generated convincing results. While these immunosuppressive medications have an effect on T cell dynamics, none of them profoundly deplete T cell populations, so it is not surprising that they have been inconclusive or even contraindicated in viral induced autoimmune disease. These reviews also stress that immunosuppression should not be given unless the patient has been determined to be virus free, a view that appears to represent a consensus statement and contrary to Woodruff’s classic study (61). This is also a contradiction to the numerous animal studies that found that myocarditis was related to the manifestations of T cell mediated injury and not active infection or the presence of viral genome in the heart. The virus infection may initiate the autoimmune process but might otherwise be irrelevant to the outcome. The caveat might be that the murine studies focus on the acute pathophysiology of the disease which, in the human, presents as fulminant myocarditis while the non-T cell depleting therapies might be more appropriate for a chronic disease state.

### Type II Hypersensitivity in COVID-19

Viruses may induce an antibody mediated autoimmune process (Type II hypersensitivity). The B cell lineage produce these antibodies and myocarditis animal models point to the considerable impact that B cells and their resultant antibodies have in the pathophysiology (77–83). Autoantibodies may be produced by an immune response to viral antigens that then cross-react with self-antigens in a process called molecular mimicry. Viruses may also injure myocardial cells and cause them to release self-antigens to which new autoantibodies may form (83). Antibodies may be made against cell surface antigens as well as intracellular and even intra-mitochondrial antigens (79, 82). Making the diagnosis of antibody mediated myocarditis can be challenging in that immunohistochemical studies may be difficult to obtain in community hospitals and the endomyocardial biopsy may be normal. While autoantibodies are well documented in some patients with lymphocytic myocarditis, it is probable, that autoantibodies play a role in the pathophysiology of Kawasaki disease associated with COVID-19. Autoantibodies are a product of the adaptive immune system wherein T cells and B cells interact to form antibody. A brief review of antigen recognition and antibody production and immunologic memory may reveal new strategies to remove harmful antibodies in COVID-19.

When SARS-CoV-2 enters the body via the respiratory system or gastrointestinal tract it is likely to be taken up by antigen presenting cells (APCs), e.g., dendritic cells, macrophages, epithelial cells, and B cells. Dendritic cells that capture the virus become activated and express major histocompatibility antigens (MHCs) that present small fragments of virus polypeptide in a groove in the MHC. They also present co-stimulatory molecules on their surface that work to activate naïve T cells. The dendritic cells travel to lymph nodes where they will meet naïve T cells, a few of which are specific for the MHC-peptide complex. In response to the MHC-peptide recognition, the naïve T cells then become activated, secrete cytokines, undergo clonal expansion, and differentiate into effector and memory subsets. The effector T cells work to eliminate the virus while memory T cells are long lived, move out of the lymph nodes to sites where they may encounter viral antigens and rapidly respond to subsequent encounters with the virus. There are two major types of effector T cells, CD4^+^ T cells, or helper T cells, that help B cells to make antibodies to the SARS-CoV-2 virus and CD8^+^ T cells that are able to kill virus infected cells.

The SARS-CoV-2 virus will also attach to a B cell receptor that is bound to the membrane of a mature naïve B cell. These cells express CD20 on their surface and are usually located in a lymph node that drains the respiratory or gastrointestinal tract. The B cell, with help from other cells and cytokines may then become activated and it can travel to the germinal center part of the lymph node and engage with a CD4^+^ helper T cell. The virus, on the surface of the B cell is internalized, broken apart and a peptide fragment from the virus is then presented in an MHC II molecule on the surface of the B cell. The SARS-CoV-2 activated B cells then meet with the independently activated SARS-CoV-2 CD4^+^ T cells and the CD4^+^ T cells then recognizes the MHC II-peptide complex on the B cells. Once a B cell becomes further activated by a CD4^+^ T cell, it loses its CD 20 surface molecule, and it may become a short-lived plasma cell and generate antibody, or it can enter a geminal center where it can undergo somatic hypermutation with affinity maturation and isotype switching. The B Cell can then differentiate into a plasmablast and secrete antibody or can become a memory B cell, which is long lived and has CD27 on its surface. Some plasmablasts may find a survival niche in the bone marrow and become a long-lived plasma cells (84).

The elimination of viral induced autoantibodies requires reduction or elimination of the plasmablasts and the short-lived plasma cell populations responsible for making the antibody. It also involves the reduction or elimination of memory B cells sensitized to autoantigens that are primed to become high affinity plasmablasts or plasma cells upon re-exposure to autoantigens. The population of pre-sensitized memory T cells, that are capable of re-stimulating B cells to differentiate into plasmablasts and autoantibody producing plasma cells, also need to be eliminated. A successful plan to eliminate production of autoantibody must address two of the three systems for immunologic memory: memory T cells and memory B cells. The third system of immunologic memory, the long-lived plasma cells that are held within protective niches within the bone marrow, probably doesn’t need to be addressed since COVID-19 is an acute disease and there is not enough time for long-lived plasma cells to compete for a survival niche.

Thymoglobulin has T cell depleting activity via antibodies to several T cell antigens including CD3, CD4, CD8, CD28, and CD 45 (69, 71). It also has antibodies to HLA-ABC and HLA-DR and the B cell specific surface proteins CD19, CD20, CD80, and CD 40 and the plasma cell surface protein CD138 (71). It has been shown to induce apoptosis of stimulated B cells and plasma cells *in vitro*, but does not delete plasma cells from the human spleen (85).

Rituximab, an anti-CD20 monoclonal antibody [Rituxan (Rituximab) Genentech, South San Francisco, CA, United States], was first approved for B cell lymphomas and has found efficacy in treating multiple sclerosis, rheumatoid arthritis, and other autoimmune diseases. It binds to human CD20 on mature naïve B cells and depletes B cells in circulation for 4–12 months (86). In a murine model it was shown to decrease autoreactive short-lived plasma cells thus reducing serum autoantibodies while not affecting total antibody titers (87). Plasma cells in bone marrow niches do not display CD20, so antibody titers from these cells are not affected.

RATG and rituximab have been used together in highly sensitized kidney transplant recipients who then underwent splenectomy as part of a protocol (88). Analysis of the splenic tissue revealed that the RATG and rituximab combination resulted in a decrease in CD27^+^ cells, the phenotype of the memory B cells, when compared with patients given rituximab and IVIG, or IVIG alone, or control patients. These findings support the use of RATG and rituximab together in patients with autoreactive antibodies wherein the duration of the disease has been short enough so that long lived plasma cells have not yet been generated. More work in this area is needed confirm clinical efficacy in viral mediated Type II hypersensitivity reactions.

IVIG is a successful therapy alone or in combination with steroids as mentioned above in the discussion of KD. IVIG is thought to work by a number of pathways including anti-idiotypic antibodies, inhibition of cytokine gene activation, anti-T cell receptor activity, anti CD4^+^ activity, stimulation of cytokine receptor antagonists, inhibition of complement activity, and Fc mediated interactions with antigen presenting cells to block T cell activation (89). Recent work reveals that these mechanisms are possibly erroneous. Studies in children with ITP in 1993 revealed that infusion of Fc fragments provided the anti-inflammatory properties of IVIG (90). The anti-inflammatory properties of IVIG can now be attributed to Fc sialylation of IgG (91–93). Immunoglobulins are glycoproteins and a single N-linked glycan is found at Asn297 in the Fc fragment. This covalently linked complex glycan is composed of a biantennary heptapolysaccharide containing *N*-acetylglucosamine and mannose and two terminal sialic acid residues (93). Further modifications of this carbohydrate structure are common and over 30 different glycans have been identified at this one site and glycosylation of IgG is mandatory for FcγR binding. The total anti-inflammatory activity of IVIG depends on the sialylation of the IgG Fc fragments and this represents only 5% of the IgG pool. The small amount of sialylated IgG in IVIG explains why large doses are required for its anti-inflammatory effects while much lower doses are required to treat hypogammaglobulinemia. The anti-inflammatory activity of sialylated IgG is also dependent of FcγRIIB expression on immune cells in that when this receptor is deleted, IVIG loses its anti-inflammatory activity. The receptor for the sialylated IgG is not yet identified, but there is a mandatory interaction through the FcγRIIB receptors ITIM motif that leads to a suppression of cellular activation. A decrease in sialylated IgG, acting through an unidentified receptor, may be the mechanism by which the “rebound phenomenon” occurs after plasmapheresis. Plasmapheresis, by decreasing sialylated IgG, leads to the up-regulation of antibody synthesis in plasma cells and the rapid return of antibodies. Fortunately for children with KD, IVIG is enough of an immunosuppressant to decrease the harmful autoantibodies for many patients.

### Preventing and Ameliorating COVID-19

Multiple efforts are underway to develop vaccines for COVID-19 with hopeful comments appearing in the lay media for a successful vaccine within 6 months. Most countries have instituted some form of “social isolation,” mask wearing, and restrictions on travel. These are effective measures, but the virus is challenging all societies.

Ameliorating COVID-19 in populations at high exposure risk, e.g., healthcare workers, is an area of active investigation with the use of interferons (INFs). As explained earlier, the SARS-CoV-2 virus inhibits the production of type I and type III INFs in cell cultures. When exogenous INFs are added to these SARS-CoV-2 infected cell systems, viral replication is inhibited, signifying that while the production of IFNs are inhibited, its protective functions within the cell are not (94–97). The use of exogenous IFNs is especially attractive since some are already available and have been used clinically in other diseases and therefore have an established risk profile. PEGylated IFN-α was the standard of care for Hepatitis C virus until introduction of more effective drugs and IFN-λ has undergone phase II clinical trials for Hepatis C virus. The INFs are also attractive in that they may be used in a preventive manner providing increased safety for healthcare workers and others in high exposure risk occupations and for treatment of patients in early stages of the disease.

## Discussion

A key issue with therapy for COVID-19 is whether the pathophysiology is primarily infective or autoimmune. The fact that over eighty percent of patients infected have no symptoms or mild symptoms intimates that the disease response is secondary to host factors. Viral induced Type II and Type IV hypersensitivity reactions are well described, and COVID-19 fits with other viral induced autoimmune disease pathologies. The SARS-CoV-2 virus induces the same pathological scenarios as many other viruses, the unique feature is that it just does it more often.

Review of the basic science literature may help to identify therapies that might be more efficacious than others. The old “blunt tools” of corticosteroids, IVIG, cytotoxic drugs, interferons, and depletion or ablation drugs, i.e., RATG, still have their place while “Precision medicine” machines the perfect monoclonal antibody to block an offending cytokine or pathway (98). There are currently 2,713 studies listed on clinicaltrials.gov to investigate drugs and other aspects of the COVID-19 epidemic. Review of all the immunomodulating therapies is beyond the scope of this article.

The cytokine storm syndrome of COVID-19 is typified by a loss of a negative feedback loop within the immune system that then results in a positive feed forward system of exponentially increasing overproduction of inflammatory cytokines (98). While there are probably several initiating pathways to induce a cytokine storm, T cell mediated stimulation is well documented. T cell depleting medications have shown efficacy in sHLH, MAS and myocarditis and should be explored for COVID-19. The marked lymphopenia should not be a contraindication for T cell depletion since the population of T cells has most likely moved to the periphery and are no longer residing in the blood. T cells in the blood are only on their way to work. Given the efficacy of other medications like steroids, IVIG, and some of the numerous monoclonal agents, T cell depletion would be used for those patients displaying a rapid decline secondary to sHLH or MAS but before they are moribund. Patients with acute cardiac injury patterns, impending intubation, early shock, and patients who have failed other forms of therapy are potential candidates. To date, case reports using T cell depleting therapies for COVID-19 have not been reported and should be considered an area of investigation. The combination of T cell depletion and anti-CD20 antibody for severe Type II hypersensitivity reactions not amenable to IVIG and steroids seems reasonable. None of the immunosuppressive medications have any efficacy in removing the offending virus and antiviral therapies should be employed to decrease the time of viral shedding. Remdesivir or other antivirals should probably be considered as part of a treatment regimen in COVID-19.

## Author Contributions

The author confirms being the sole contributor of this work and has approved it for publication. TI designed and wrote the manuscript.

## Conflict of Interest

TI was employed by Tim Icenogle MD, PLLC.
